# Automated Image Analysis for the Detection of Benthic Crustaceans and Bacterial Mat Coverage Using the VENUS Undersea Cabled Network

**DOI:** 10.3390/s111110534

**Published:** 2011-11-04

**Authors:** Jacopo Aguzzi, Corrado Costa, Katleen Robert, Marjolaine Matabos, Francesca Antonucci, S. Kim Juniper, Paolo Menesatti

**Affiliations:** 1 Instituto de Ciencias del Mar (ICM-CSIC), Paseo Marítimo de la Barceloneta 37-49, Barcelona 08003, Spain; 2 Agricultural Engineering Research Unit of the Agriculture Research Council (CRA-ING), Via della Pascolare 16, 00015, Monterotondo scalo, Rome, Italy; E-Mails: francescaantonucci@hotmail.it (F.A.); paolo.menesatti@entecra.it (P.M.); 3 School of Earth and Ocean Sciences and Department of Biology, University of Victoria, P.O. Box 3065 STN CSC, Victoria, BC V8W 3V6, Canada; E-Mail: katleenr@uvic.ca; 4 NEPTUNE-Canada, University of Victoria, P.O. Box 1700 STN CSC, Victoria, BC V8W 2Y2, Canada; E-Mails: mmatabos@uvic.ca (M.M.); kjuniper@uvic.ca (K.J.)

**Keywords:** cabled observatory, automated image analysis, squat lobster (*Munida quadrispina*), bacterial mat (*Beggiatoa* spp.), Scale-Invariant Feature Transform (SIFT), Fourier Descriptors (FD), Partial Least Square Discriminant Analysis (PLSDA), percentage of coverage, fractal dimension

## Abstract

The development and deployment of sensors for undersea cabled observatories is presently biased toward the measurement of habitat variables, while sensor technologies for biological community characterization through species identification and individual counting are less common. The VENUS cabled multisensory network (Vancouver Island, Canada) deploys seafloor camera systems at several sites. Our objective in this study was to implement new automated image analysis protocols for the recognition and counting of benthic decapods (*i.e.*, the galatheid squat lobster, *Munida quadrispina*), as well as for the evaluation of changes in bacterial mat coverage (*i.e.*, *Beggiatoa* spp.), using a camera deployed in Saanich Inlet (103 m depth). For the counting of *Munida* we remotely acquired 100 digital photos at hourly intervals from 2 to 6 December 2009. In the case of bacterial mat coverage estimation, images were taken from 2 to 8 December 2009 at the same time frequency. The automated image analysis protocols for both study cases were created in MatLab 7.1. Automation for *Munida* counting incorporated the combination of both filtering and background correction (Median- and Top-Hat Filters) with Euclidean Distances (ED) on Red-Green-Blue (RGB) channels. The Scale-Invariant Feature Transform (SIFT) features and Fourier Descriptors (FD) of tracked objects were then extracted. Animal classifications were carried out with the tools of morphometric multivariate statistic (*i.e.*, Partial Least Square Discriminant Analysis; PLSDA) on Mean RGB (RGBv) value for each object and Fourier Descriptors (RGBv+FD) matrices plus SIFT and ED. The SIFT approach returned the better results. Higher percentages of images were correctly classified and lower misclassification errors (an animal is present but not detected) occurred. In contrast, RGBv+FD and ED resulted in a high incidence of records being generated for non-present animals. Bacterial mat coverage was estimated in terms of Percent Coverage and Fractal Dimension. A constant Region of Interest (ROI) was defined and background extraction by a Gaussian Blurring Filter was performed. Image subtraction within ROI was followed by the sum of the RGB channels matrices. Percent Coverage was calculated on the resulting image. Fractal Dimension was estimated using the box-counting method. The images were then resized to a dimension in pixels equal to a power of 2, allowing subdivision into sub-multiple quadrants. In comparisons of manual and automated Percent Coverage and Fractal Dimension estimates, the former showed an overestimation tendency for both parameters. The primary limitations on the automatic analysis of benthic images were habitat variations in sediment texture and water column turbidity. The application of filters for background corrections is a required preliminary step for the efficient recognition of animals and bacterial mat patches.

## 1. Introduction

The history of humanity mostly developed along coasts [1] and continental margin areas are therefore experiencing increasing human pressure. Marine ecosystems in these areas are not only exposed to contamination, but also to increasing fishing activity, which is progressively moving seaward to deeper waters [2]. These dynamics of these hydrodynamically changeable environments [3] and their marine ecosystems are poorly described as are their responses to external human influences. The lack of reliable oceanographic sensor technology for long-term continuous observations has been a major obstacle to improving our understanding of physical and biological processes in the oceans [4]. New tools are therefore required for the remote monitoring and management of continental margin areas at depths from coastlines to the continental slopes that lead to the deep-sea. Such tools need to be deployed in situ and allow remote access, continuous, long-term, and high-resolution acquisition of data [5–7]. Monitoring should not only encompass oceanographic and geophysical or chemical parameters (*i.e.*, the habitat) but it should also include bio-data related to abundance, composition and activities of species inhabiting the seafloor and overlying water column [8,9]. Newly-developed cabled observatory technologies offer a promising solution to the need to acquire long time series of data suitable for ecosystem modelling and ecosystem-based management [10–15].

Presently, the design, manufacture and deployment of sensors for cabled observatories are biased toward the measurement of habitat variables, while sensor technologies for biological community characterization through species identification and individual counting are less developed [4]. Sensors that quantify photosynthetic pigments, dissolved nitrate salts, and dissolved oxygen concentrations can be considered to provide indirect measurements of biological activity in the ocean [16]. However, sensors and sensor systems that directly quantify the biological activity of animals, populations, and species in terms of presence/absence and behaviour are lacking [17]. Underwater imaging techniques that use still cameras, video and both passive (hydrophones) and active (sonar) acoustic devices are probably the best current tools for remote biological observations in the ocean [18,19]. While active acoustic instruments are still chiefly used for water column measurements, video and still cameras are best suited for study of biological communities on the seafloor [20].

The VENUS cabled network [21], located on Vancouver Island (British Columbia, Canada [22]), supports digital still and video camera systems. One of these cameras has been located at 103 m depth on the edge of the main basin of Saanich Inlet, a fjord at the southern end of the island. This multi-sensor platform allows the remote, continuous, and real-time video observation of seafloor organisms together with physical and chemical variables (e.g., temperature, salinity, pressure, dissolved oxygen and nitrates [21], Its camera can be remotely controlled, hence providing a unique suite of instruments for interdisciplinary studies on benthos in relation to key environmental variables [18,23]. Quantitative biological data extraction from VENUS photos and video sequences is limited by the lack of reliable automation techniques in frame/footage processing. However, this platform provides a very interesting and challenging context for biosensor development to improve our capability for automated animal tracking and classification.

Saanich Inlet is a naturally hypoxic/anoxic fjord, with the depth of the anoxic layer varying throughout the year depending on water renewal events and oxygen depletion by organisms [24]. The VENUS camera was strategically deployed slightly up slope from the anoxic waters, within a zone of fluctuating hypoxia. This setting is ideal to study population movements in relation to changing oxygen concentrations and understand how those changes affect the community dynamics. For example, two community elements are of broad ecological interest; the squat lobster (galatheid crab) *Munida quadrispina*, the feeding and locomotory activities of which disturb surface sediments [25], and filamentous bacterial mats that intermittently cover the sediment surface, are indicators of ecosystem anoxia and hence stress status. Bacterial mats are formed by *Beggiatoa* spp., a sulphide oxidising organism that inhabits the interface between anoxic and oxic conditions. In shallow waters, these bacteria have been observed to undertake diel cycles of burying within the substratum in response to changing redox conditions [26]. The mats occupy extensive area of seafloor below 100 m depth in the Inlet, varying in coverage with the annual cycle of anoxia [27]. *Munida* are abundant and especially well adapted to the low and fluctuating oxygen concentrations found at these depths in Saanich Inlet [28].

While camera systems are commonly included in multi-sensor platforms deployed on the seafloor, their use is still chiefly too descriptive. In this study, our objective was to develop new automated image analysis protocols customized for the VENUS deep camera site in Saanich Inlet. We targeted *Munida* and the bacterial mats for automated quantification. For the bacterial mats, our goal was to automatically estimate variations in coverage of the seabed.

## 2. Materials and Methods

### 2.1. The VENUS Cabled Observatory Video Camera and Image Acquisition

In this study we used still imagery from the VENUS cabled observatory C-MAP Cyclops underwater camera, modified with a Olympus^®^ C8080 wide zoom (8 Megapixels, f2.4, 5x optical zoom) in order to acquire digital still photos of the surrounding benthic area at a high resolution (3,264 × 2,448 pixels; 72 dpi). The camera was mounted on a small, ROV-deployable tripod together with a Sidus SS209 pan & tilt unit (±/− 90° tilt and ±/− 180° pan). An Ikelite 200 Ws flash was used in all image acquisition sessions. To acquire imagery users logged in remotely to the shore-station computer that controlled all camera and accessory functions.

For the development of the automated image analysis protocol for *Munida* counting, we acquired 100 digital photos at hourly intervals from 2 to 6 December 2009, beginning at 8:00 am and ending at 10:00 am. An oblique angle of 45° was chosen for photo acquisition (*i.e.*, at fixed pan/tilt camera coordinates) in order to encompass a seabed area of approximately 90 × 1,200 cm.

For bacterial mat estimation, image acquisition occurred with the camera oriented vertically down, picturing a seabed area of approximately 30 × 40 cm. The white bacterial mats were highly reflective and a vertically down camera angle provided a more uniform lighting field than did oblique photos.

### 2.3. Image Analysis Protocol for *Munida*

**Figure 1.**
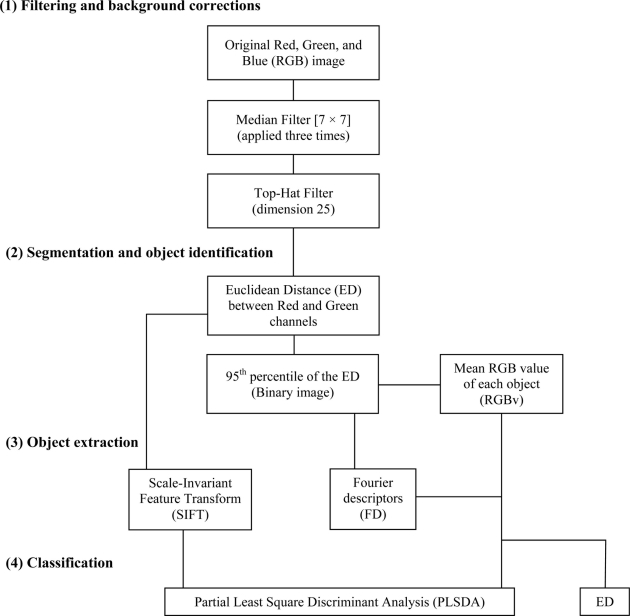
Flowchart detailing the customized image analysis protocol for the automated detection of squat lobsters (*Munida quadrispina*) with the VENUS cabled observatory imaging system.

An automated image analysis protocol was developed in MatLab 7.1. A flow-chart depicting the consecutive steps used for the tracking and classification of is presented in Figure 1. Principal problems in relation to the automation of animal detection were: (i) uneven seabed illumination by the platform lights; (ii) temporally variable background texture; (iii) water column turbidity; and finally, (iv) the presence of other, non-target species.

**Figure 2.**
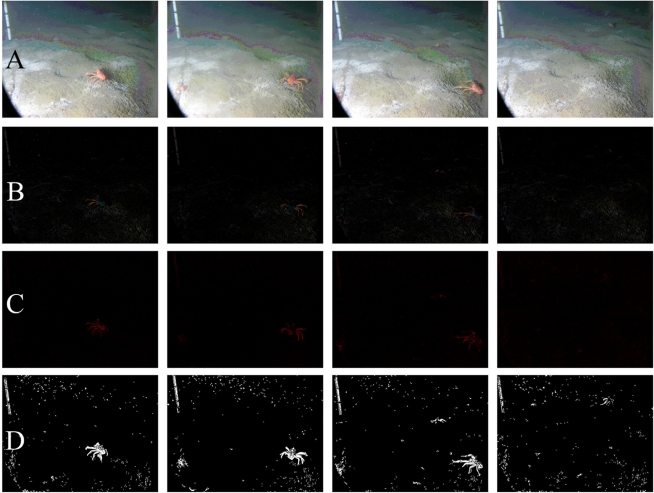
Example of processing of four temporally consecutive images (taken from 8:00 to 12:00 on 2 December 2009) for the automated identification and counting of the squat lobster (*Munida quadrispina*) at VENUS cabled observatory. (**A**) original RGB image, in which the metric bar appears (metric bar is on the upper left; black mark units are speared of 10 cm); (**B**) the same image after Median filtering and background correction with Top-Hat; (**C**) ED’s between R and G channel calculated for each pixel of the Top-Hat filtered-image; (**D**) segmentation using a threshold value corresponding to 95th percentile of ED.

Figure 2 shows an example of the digital outputs for the processing steps indicated in the flowchart of Figure 1. Filtering and background corrections were preformed on original Red, Green, and Blue (RGB) digital images [Figure 2(A)]. In order to reduce the overall noise, images were filtered three times by means of a Median Filter [7 × 7]. Then, the uneven illumination conditions were adjusted by applying a Top-Hat Filter to the background (*i.e.*, dimension 25) [Figure 2(B)]. The name “Top-Hat” originates from the shape of the filter, which is a rectangle function, when viewed in the domain of the frequencies in which the filter is constructed [29]. This filter is highly efficient for the enhancement of small objects in busy backgrounds, when a background image with no animals as reference is not available for frame subtraction. It uses a morphological method to extract the background “grain” associated with an image resulting from the subtraction between the original and filtered images. The morphological filter dimension is important. At small scales, the filter enhances particularities, while as scale increases, the filtering efficacy is reduced and becomes more general. Therefore, we selected its relative dimension by considering the size relationship between the object (*i.e.*, *Munida*) and ROI size.

Segmentation and object identification (see Figure 1) were carried out on the Top-Hat filtered images. The Euclidean Distance (ED) between R and G channels was calculated for each pixel [Figure 2(C)]. This image was segmented into binary form, by applying a threshold value corresponding to the 95th percentile of the ED distribution [Figure 2(D)] and by using a Scale-Invariant Feature Transform (SIFT) (see below). This final digital product was used to identify and to extract the mean RGB value (RGBv) of each object from the original image and the Fourier Descriptors (FD).

Object identification was based on two different approaches (see Figure 1). Animal bodies were identified according to FD analysis or alternatively with SIFT. The outputs of both methods were compared in terms of efficiency.

FD were obtained from the Fourier transformation of a complex function representing *Munida* outlines in pixel coordinates [30]. FD analysis is based on the scalability of a curve as a closed contour describing the shape of an organism: by varying the number of Fourier coefficients used, different levels of approximation of the Fourier function to the animal profile can be obtained [31]. FD values for *Munida* were normalized and transformed to be size, orientation, and translation independent. Sixty descriptors were used, corresponding to 128 coefficients. Only objects having a pixel area comprising between 200 and 150,000 pixels were considered for this analysis in order to eliminate random contingent noise (*i.e.*, turbidity, debris and non target benthic species).

**Figure 3.**
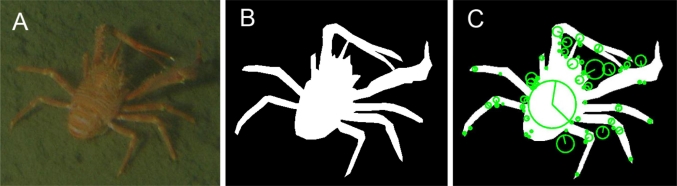
Example of the SIFT processing for the identification of a squat lobster (*Munida quadrispina*) within a digital still image at VENUS cabled observatory. Sequences of digital image products obtained by the automated processing are: (**A**) The original RGB image; (**B**) Binarized mask of the extracted animal; and finally, (**C**) SIFT features extracted in green (*i.e.*, those considered as belonging to the object had the centre of the features located within the white mask area of B).

SIFT is an algorithm developed in the computer vision domain for detecting and describing local features in digital images [32]. The convolved images are grouped by octave (*i.e.*, an octave corresponds to doubling the value of scale space). After a preliminary and technical overview, we established the following states for the SIFT parameters: (i) the number of octaves was equal to 9; (ii) the number of levels per octave of the Difference of Gaussian scale space was equal to 3; (iii) Peak selection thresholds also equalled 3; and finally, (iv) The Non-edge Selection threshold was set at 7. The technical processing for *Munida* identification is detailed in Figure 3. For each extracted feature, 128 descriptors were used as variables in the analyses from that moment on. In our case, we considered tracked displacing objects to belong to the *Munida* category (*i.e.*, hence counting for one animal), if these had a number of features greater than 6, with a pixel distance closer than 500.

Object Classification was the last step of the analysis (see Figure 1). This was carried out by using the tools of morphometric multivariate statistics. Two different methods were applied in order to classify each new tracked animal within the *Munida* species as an *a priori* category: Partial Least Square Discriminant Analysis (PLSDA) and ED.

PLSDA is a soft-modelling multivariate statistical approach that allows identification of correlated pairs of linear combinations (*i.e.*, singular vectors) between two blocks of variables [33]. The result of this technique is the construction of a predictive model made by many and highly collinear factors [34].

PLSDA analysis was conducted on both the SIFT features plus the RGBv for each object and Fourier Descriptors (RGBv+FD) matrixes. For both datasets a preliminary training procedure was applied, in order to build the model of reference. This training procedure was accomplished by using: (i) 38 images of *Munida* (*i.e.*, 2219 features within and 880 outside the animals’ body; see Figure 3) for SIFT matrix; (ii) 4800 objects (*i.e.*, 57 *Munida* and 4,743 other extraneous objects) for the RGBv+FD. PLSDA looked for correlations among the matrices values (x-block; 128 matrix for SIFT and 3+128 matrix variables for RGBv+FD) and the y-block, which was composed by two dummy (*i.e.*, categorized) variables. Dummy variables were categorized as (1) and (2) if “belonging” or “not belonging” to an extracted *Munida* body, respectively. The X-block was pre-processed with the ‘mean centre’ procedure.

After tracking animals, PLSDA allows an evaluation of its classification performance, indicating the modelling efficiency in terms of sensitivity and specificity of the chosen parameters. The sensitivity is the percentage of the samples of a category accepted by the class model. The specificity is the percentage of the samples of the categories different from the modelled one, which are rejected by the class model. Generally, the residual errors show a decreasing trend in the calibration phase (*i.e.*, Root Mean Square Error of Calibration, RMSEC) and an increasing trend in the validation phase (*i.e.*, Root Mean Square Error of Cross-Validation, RMSECV; [35]).

For FD and SIFT analyses, each group was subdivided into two sub-groups: 70% of objects for the class modelling and validation, and 30% of objects for the independent test, optimally chosen with the ED based on the algorithm of Kennard and Stone [36]. This algorithm selects objects without an *a priori* knowledge of a regression model (*i.e.*, the hypothesis is that a flat distribution of the data is preferable for a regression model; [37]).

The RGBv+FD matrix (*i.e.*, 3+128 variables) was also classified using the ED approach, being these distances equal to the square root of the sum of the squared difference for each dimension (*i.e.*, variable). ED’s are extremely sensitive to the scales of the variables involved. In geometric situations, all variables are measured in the same units of length. For this reason, we computed two different ED thresholds for RGBv values and FD.

In accordance with current standards [30,38,39], an efficiency test was carried out in order to evaluate the performance of the different methodologies employed for the automatic object classification (see Figure 1). Error estimation was calculated on all the 100 images in comparison with visual results provided by a trained operator (*i.e.*, manual counting). Error typologies were categorized into object identification and object classification. The occurrence of the two different errors was hence determined: misclassification when a *Munida* was present within the frame but not detected (*i.e.*, Error Type-1); and wrong classification when a *Munida* was detected when not present in the picture (*i.e.*, Error Type-2). Also, a time series of visual count outputs from our automated protocol for the different combinations of analytic methods (*i.e.*, RGBv+FD and PLSDA; SIFT and PLSDA; RGBv+FD and ED) were graphed together, in order to obtain a visual estimation of their efficiency.

### 2.4. Bacterial mat Coverage Estimation

**Figure 4.**
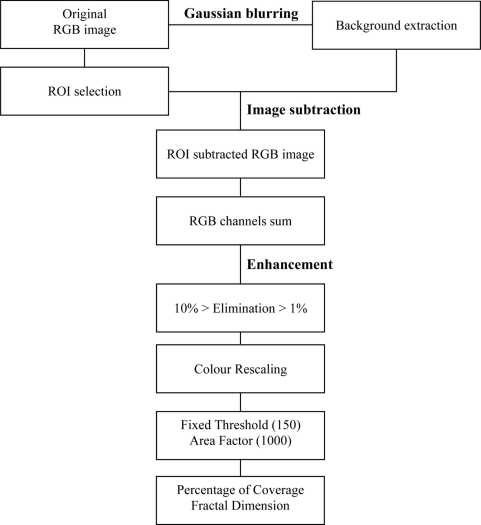
Flowchart detailing the different steps of the protocol for the automated estimation of bacterial mat (*Beggiatoa* spp.) coverage in images from the VENUS cabled observatory.

We developed an automated image analysis protocol for bacterial mat coverage estimations in MatLab 7.1. Our aim was to compute the Percentage of Coverage and Fractal Dimension. A flow chart detailing the consecutive steps of image treatment is presented in Figure 4.

A total of 52 images were considered as suitable for the analysis. Benthic species commonly present in the area caused a disturbance effect, either covering the ROI or moving sediment around. These species were chiefly the slender sole (*Lyopsetta exilis*), the Pacific herring (*Clupea pallasii*), and *Munida* itself [23].

Matabos *et al.* [23] describe the estimation of bacterial mat coverage by measuring how the mats filled space in images of the seafloor. This type estimation can be carried out by computing the Percentage of Coverage (*i.e.*, the surface covered by bacterial mats) and the Fractal Dimension, the latter being a measure of how completely the bacterial mats fill the space at increasingly smaller scales (*i.e.*, the covering geometrical complexity). Fractal analysis can be used to describe the occupation of space by biological forms such as the branching patterns in tree roots or spatial structure in mussel beds (reviewed by reference [40]). Both parameters are usually estimated in a semi-automated fashion, using software that requires the manual identification of areas to be analyzed in digital images.

The main operative problems in establishing a successful protocol were related to the uneven background illumination. Additionally, the analysis was complicated by: (i) variable and fragmented background, and (ii) fish-eye image distortion due to the camera lens.

**Figure 5.**
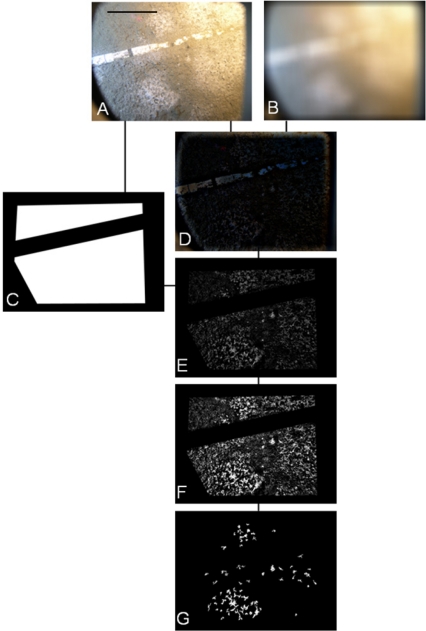
Example of the automated processing of an image with bacterial mats (*Beggiatoa* spp.) as acquired by VENUS cabled observatory imaging system(scale bar: 10 cm): (**A**) Original image where the metric bar appears as semi-burrowed in the centre; (**B**) Background extraction with the Gaussian Blurring; (**C**) ROI definition (white areas); (**D**) Subtraction of the original image (in A) with its background (in B); (**E**) Sum of the RGB channels (ROI-selected); (**F**) Enhancement and rescaling; and finally, (**G**) Fixed thresholding (150) and small (<1,000 pixels) area eliminations. The Percentage of Coverage and the Fractal Dimension were calculated in the final digital product (in G).

The different outputs of automated image processing are reported as an example in Figure 5. As a first step in processing, a ROI was preliminarily selected in each original RGB image [2,448 × 3,264; Figure 5(A)]. For each image the background was extracted applying a Gaussian Blurring Filter procedure [41] [Figure 5(B)]. The ROI was then enhanced and preserved as common for all processed images [Figure 5(C)]. This low-pass filter attenuates high frequency signals by applying a Gaussian function to a squared pixel kernel (*hsize*). The standard deviation of this function (*sigma*) was also defined. Both values were empirically determined in a ten images training set, as follows:

(1) $$\mathrm{hsize}=10\%\sqrt{\mathrm{Area}\;\mathrm{img}}$$

(2) $$\mathrm{sigma}=10\%\sqrt{\mathrm{Area}\;\mathrm{img}}$$

During the second step of automated processing (*i.e.*, Image Subtraction; see Figure 4), the resulting background image was subtracted to the original one within the ROI [Figure 5(D)]. The resulting RGB values were then summed [Figure 5(E)].

In order to enhance the images which varied in the distribution of their intensity, 10% smaller and 1% larger values were eliminated, by setting them as equal to the lower and higher nearest values, respectively. These values were chosen after empirical evaluation of ten images in the training set. The resulting gray scale image was then rescaled from 0 to 255 values [Figure 5(F)]. Then, a fixed threshold was set up on the resulting digital product and objects smaller than 1000 pixels were eliminated (*i.e.*, the Area Factor), resulting in the final black and white image [Figure 5(G)].

The Percentage of Coverage by the bacterial mats in this image was calculated, together with the Fractal Dimension. This last parameter was estimated using the ‘box-counting method’ [42]. In order to create “square” boxes, the image was automatically resized to a square dimension such that the length, measured in number of pixels, was of a power of 2. This allowed for the square image to be equally divided into four quadrants and each subsequent quadrant could be then re-divided into four quadrants, and so on. The number of boxes containing “black” pixels was noted as a function of the box-size (*i.e.*, the length of box). The natural log of all these points were calculated and plotted. A linear interpolation was calculated and the slope of the line was estimated as the Fractal Dimension value.

The efficiencies in the automated computing of both Percentage of Coverage and Fractal Dimension were also compared with results generated using the software Image J (National Institutes of Health, USA; http://rsbweb.nih.gov/ij/). The latter requires the manual processing of images for: (i) binarization, in order to enhance white bacterial patches against the grey sediment background; and (ii) the estimation of parameters of interest by the Fractal Box Counter (*i.e.*, a variable number of boxes of pixels sizes equals to 2, 3, 4, 6, 8, 12, 16, 32, and 64 are manually overlaid on the image [23]).

## 3. Results

### 3.1. The counting of Munida

We identified a total number of 103 squat lobsters from all images by manual counting. By comparison, the automated image analysis protocol showed different efficiencies, depending on the terminal processing steps used (see Figure 1 for reference), which were applied in parallel on all images. RGBv+FD and PLSDA overestimated animals up to 172 positive identifications. Conversely, both SIFT and PLSDA as well as RGB+FD and ED, subestimated *Munida* counts number to a different extent (65 and 12, respectively). The results of PLSDA models on SIFT and RGBv+FD matrices are reported in Table 1. For both methods high percentages of correct classification were possible for the model and the test set, as well as high values of efficiency parameters (*i.e.*, specificity and sensitivity). We also observed low percentages of RMSEC (Table 1) and classification error (Table 2).

**Figure 6.**
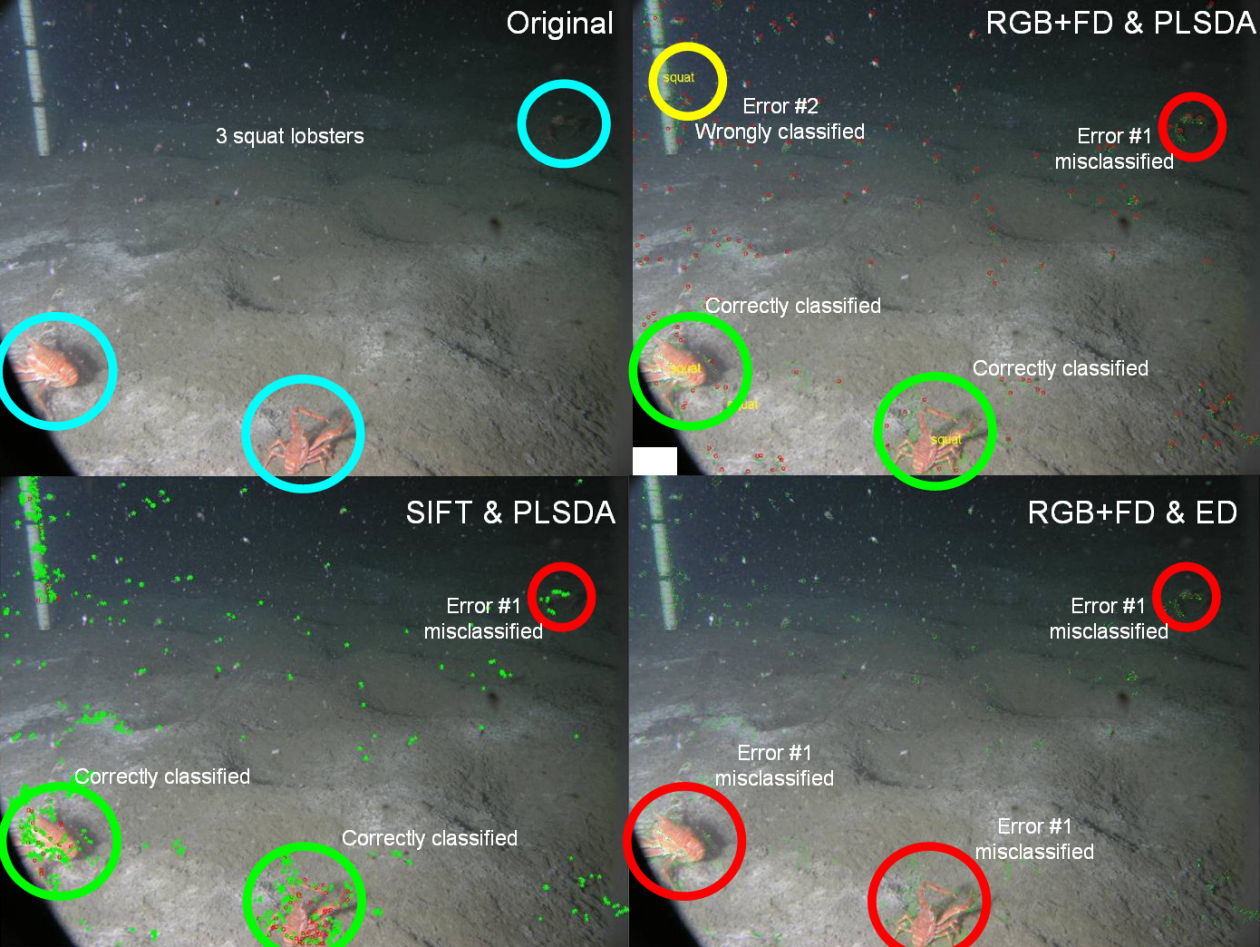
Example of different error typologies encountered during automated identification of squat lobsters (*Munida quadrispina*) in digital images taken at VENUS cabled observatory. An original image containing three individuals is presented along with different automated classification outputs in relation to the three different processing methods (see Figure 1): RGBv+FD and PLSDA; SIFT and PLSDA; RGBv+FD and ED. Blue circles highlight the animals inside the original image. Green circles represent correctly classified lobsters. Red circles indicate non-classified but present (misclassified; Error Type-1) animals. Yellow circles indicate wrongly classified (detected but not present; Error Type-2) animals.

The SIFT approach returned better results in term of animal identifications (Table 2). The presence of animals was correctly classified in 51% of images (*i.e.*, an animal is detected instead of being not present (Error Type-2). Also, lower values (51) of misclassification (*i.e.*, an animal is present but not detected; Error Type-1) were obtained. Differently, RGBv+FD and ED returned no wrong classifications but most of the objects were misclassified (91 out of 103). Figure 6 presents examples of the different typologies of error we found by processing the same image with the three different analytical methods.

**Figure 7.**
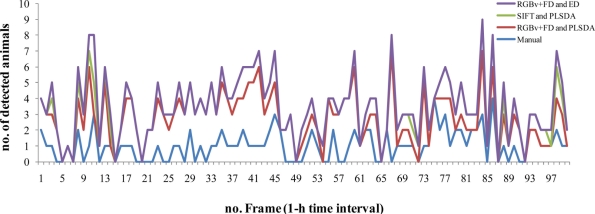
Time series of manually and automatically counted squat lobsters (*Munida quadrispina*) in digital images taken at the VENUS cabled observatory. The outputs of the three different automated methods (RGB+FD and PLSDA; SIFT and PLSDA; RGB+FD and ED3; see Figure 1) were graphed in relation to output generated by manual counting.

Time series of counted *Munida* using the different automated protocols (*i.e.*, RGB+FD and PLSDA; SIFT and PLSDA; RGB+FD and ED; see Figure 1 for processing steps details) are presented in Figure 7 as an indication of their variable performance. The automated protocols showed little difference when compared together, while this difference was always larger in relation to the manual counting. This was chiefly due to the presence of Type-2 Errors in the automated counting. Moreover, no diel (*i.e.*, 24-h based) variations in counted animals were apparent.

### 3.2. Bacterial Mat Coverage Estimation

**Figure 8.**
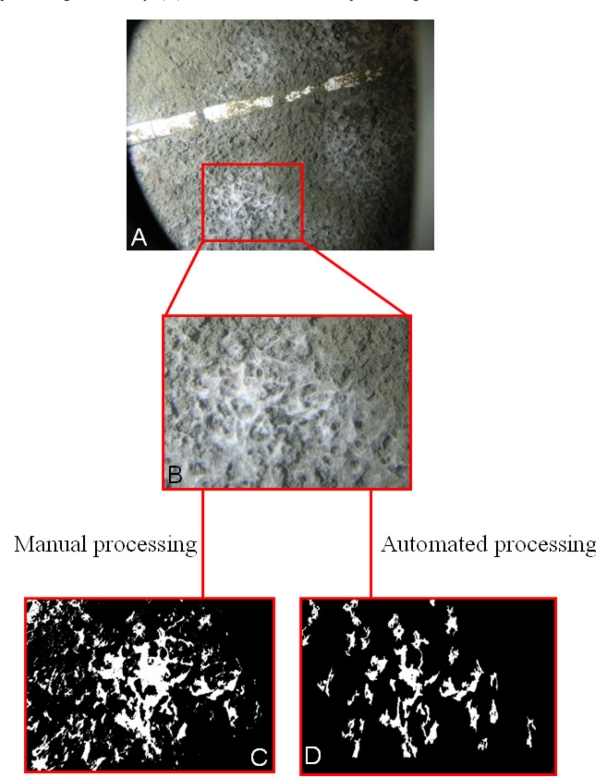
Example of a manual and automated processing of bacterial mats (*Beggiatoa* spp.): (**A**) Original image as an example; (**B**) Zoomed area; (**C**) Result of the manual processing; and finally, (**D**) Result of the automated processing.

The outputs from single digital images of bacterial mat identification are presented in Figure 8 as an example of the manual and automated processing outputs. This provides a general overview of achieved efficiency with the automation procedure proposed in Figure 4. By considering an originally acquired digital image [Figure 8(A)] and delimiting only a fraction of it [Figure 8(B)], it is possible to observe the different results of manual and automated estimation [Figure 8(C)]. Generally, the automated method extracted a smaller area with respect to the manual one due to the background correction approach.

The Pearson correlation coefficients for the Percentage of Coverage and the Fractal Dimension for the manual and automated processing were high for both, being 0.67 and 0.76, respectively, hence confirming the good accordance between the methods.

**Figure 9.**
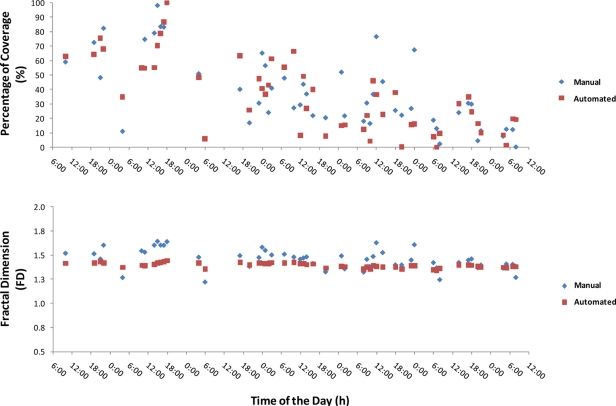
Time series of Percentage of Coverage and Fractal Dimension of bacterial (*Beggiatoa* spp.) mat as measured in manual and automatic fashion.

The manual evaluation generally gave higher estimations than the automated processing for both indicators. Time series comparing manual and automated outputs of bacterial mat assessment in terms of Percentage of Coverage and Fractal Dimension (Figure 9) confirmed the differences in both estimation methods as well as over the time, as detailed in the example of Figure 8. The overestimation tendency of the manual method is particularly evident for Fractal Dimension values, while for the Percentage of Coverage, the same seems to occur in a variable fashion with the time of the day. Out of 52 images the manual analysis overestimated the Percentage of Coverage 27 images (52.9%) in comparison to the automated protocol. There were 42 overestimation events in the case of manual counting for Fractal Dimension (80.7%). The temporal plot also provides additional insights of a more biological nature. The bacterial mat started to disappear with increasing oxygen levels (data not shown). This was observed in the area until the end of December when bacteria totally disappeared [23]. One explanation for this disappearance is a downward migration into the sediment to reach the interface between anoxic and oxic conditions, which is their required habitat [23].

## 4. Discussion

The automated detection of continental margin animals and variations in bacterial mat coverage in remotely acquired digital images represents a still underexploited means of studying marine benthic community dynamics at different levels of ecological complexity. In this study, we created two different protocols for the automated detection and counting of benthic animals (*i.e.*, the galatheid squat lobster, *Munida quadrispina*) and the estimation of bacterial mat (*Beggiatoa* spp.) coverage at a cabled observatory site.

In the case of *Munida*, we innovatively combined: image segmentation and filtering techniques for background correction; morphometric and textural tools (FD and SIFT, respectively) for shape analysis; and finally, multivariate statistical modelling (*i.e.*, PLSDA) for classification. Results showed a variable efficiency in automated protocols, when manual and automatic outputs were compared together. That variability in automated efficiency is an indication of the difficulties of working with deep-water benthic imaging products when highly variable levels of turbidity and seabed heterogeneity are encountered under artificial lighting conditions (*i.e.*, light-ON at photo acquisition). In particular, artificial lighting creates a strongly uneven background. These observations highlight the present dichotomy between the installation on cabled observatories of powerful illumination systems which are implemented chiefly for observation purposes, but which complicate the efficient extraction of quantitative bio-information in automatic fashion from footages/frames.

Still or motion video-analyses can be used to track and count individuals for different marine species in different habitat contexts and at different temporal scales [30,43–49]. While tracking can be implemented by using different protocols for image filtering such as those for pixel size, grey-levels or RGB enhancement [50,51], classification can be developed by extracting morphometric indices and analyzing their variations in different species by multivariate statistics [30]. For the counting of *Munida*, we used FDs that have already been used for animal classification purposes based on the analysis of their profile [52,53]. We implemented the classification capacity of this tool by PLSDA [50,54,55]. We firstly modelled the animal form and then we quantitatively discriminated each newly tracked individual into that pre-established morphological category. Another innovation was the implementation of a SIFT automated classification approach, by customizing it to our benthic context. This procedure returned the best results in comparisons to FD, since this method is based on specific image features that are scale-independent and more resistant to orientation and contextual illumination variations. SIFT procedures have already been applied to benthic species identification (reviewed by [55]) but species classification has not yet been carried out to date in a quantitative fashion using PLSDA.

We achieved only a moderate efficiency in animal recognition with both FD and SIFT analyses. In particular Error Type-1 (*i.e.*, animal present within the frame but not detected; misclassification) was recurrent, being mainly due to excessive turbidity, uneven artificial illumination and a non-orthogonal image plane at frame acquisition, which also created difficulties in the manual identification of animals at the distant borders of the field of view.

All these observations suggest that efficiency of the automated counting of benthos could be improved by combining photographic and acoustic imaging. In particular, acoustic imaging could be adapted to detect animals form a fixed origin (*i.e.*, a cabled platform) [56]. Although acoustic imaging does not allow species identification based on colour, it could be successfully applied to discriminate the forms of animals belonging to species with different morphologies.

Studies on *Beggiatoa* spp. mat dynamics are of importance to the characterization of the response of microbial components of benthic communities to oxygen variations. *Beggiatoa* presence is negatively correlated with dissolved oxygen levels [23]. Only a few other studies to date have experimented with automated methods for detecting bacterial mats in seafloor imagery [23,57]. Presently, digital frames are analyzed for Percentage of Coverage and Fractal Dimension estimations by software that requires the initial manual definition of a ROI [23]. We automated this step and obtained good measurement performances. In this context, we judge our effort as potentially interesting since the derived processing protocol could be successfully used to quantify mat presence in other ecologically and geologically relevant areas (e.g., hydrothermal vents and cold seeps; [58,59]), and contribute to the establishment of standard practices for the study of the relationships between geochemistry and benthic ecology [60]. Automated protocols such the one we elaborated could also be adapted to very different research fields (e.g., aerial photography, microscopic imaging or soil texture image analysis in agriculture; [61]).

Bacterial mat enhancement was successfully achieved with complex background filtering procedures prior to image binarization. The application of a low-pass filter such as the Gaussian Blurring [41] efficiently contributed to the process. This filter attenuated the high frequency signals, responsible for the background noise, hence allowing the identification of objects (*i.e.*, spots), occupying only a small sub-region of the ROI. In our use of this filter, the main problem encountered was the excessive flattening of the gray-scale chromatic range obtained after the image subtraction or after the image division (tested in this study but not considered due to the low efficacy). To solve this problem a dynamic rescaling calibrated on the specific object chromaticity was applied.

The automated coverage estimation presented problems in those digital pictures where uneven illumination overlay a variable and fragmented benthic background, depending on mat condition. Morphological filtering methods such as the Top-Hat filters are suitable for small objects in complex backgrounds containing many other objects. In those cases where only a small portion of the sediment background can be portrayed in pictures, then the modelling of that background with a smoothing function like Gaussian Blurring is required in order to reduce variability.

Deep-sea ecosystems are dynamic at temporal scales from milliseconds to millennia, and are under the influence of periodic events, in relation to geophysical cycles and seasonal processes, but also non-predictable stochastic events [16]. Cabled observatories provide an opportunity to study deep-sea communities at temporal scales which have not been previously available. High-resolution sampling will enable us to determine the relative influence of processes occurring at those different scales. The automated counting of benthic organisms may provide data sets where, at different time scales, the variation in the number of detected animals is a proxy of behavioural modulation [17]. The modulation of behaviour is ultimately indicative of an animal’s tolerance and response to habitat changes, be these changes the product of punctual human activities or cyclically occurring, as in the case of geophysical fluctuations in light intensity and internal tides. Understanding behavioural controls on animal presences in relation to habitat variability is essential to an accurate understanding of benthic community composition and quantifying and monitoring biodiversity.

Like any new technology, cabled observatories have limitations that need to be understood as new studies and applications are being planned. Here we briefly consider single examples of the main theoretical and technical limitations and possible solutions, related to the use of observatory cameras for the classification and counting of animals of different species and the quantification of long-term variations. One important theoretical problem is related to the representative power of biological data obtained from fixed point observations. This limitation can be mitigated through the use of multiple camera and sensor platforms, complementary surveys of the surrounding seafloor. Also, the cross-comparison of biological data sets form different areas will help researchers identify trends in species behavioural responses (and hence community variations) to similar geophysical cycles and habitat variations. Our example technical problem is related to the nearly unlimited power and data gathering capabilities that are the strength of cabled observatories. These features provide the means for acquiring quantities of imagery that far exceed what can be analyzed with manual techniques. The need for human observers can be reduced to a minimum if automation reaches a sufficient level of efficiency for discriminating and counting animals. As automated image analysis tools improve, they could become an embedded in observatory data management and archive systems, operating in an autonomous fashion, providing data on organism abundances in relation to measured environmental variables.

Despite contingent technical difficulties in the automation of image analysis, further research efforts are necessary in order to convert cameras into more efficient biosensors for benthic ecosystems. Compared to the present state of the art in the development of marine geo-, chemical, and oceanographic sensors, tools for the direct measurement of biological processes at the individual, population, and community levels are few in number [4,17]. Seafloor observatory cameras can produce long time series of biological data that can be related to habitat parameters recorded at similar or higher frequencies. The integrated analysis of these data sets may provide solid insights into species and communities responses to habitat changes, hence providing a means for identifying the cause-and-effect relationships that are at the base of ecosystem dynamics. Growing hypoxia in response to climate change and eutrophication is a major threat to coastal areas and continental slopes [62]. The high-resolution, long-term monitoring capabilities offered by coastal observatories like the VENUS network will help to understand the magnitude and scope of anthropogenic effects on our coastal oceans. Automated protocols for image presently under development all over the world [63,64] will be a critical component of this monitoring effort.

## Figures and Tables

**Table 1.** Results of PLSDA models on SIFT and RGBv+FD (see the text for acronyms definition). Some of the different reported parameters are: the number of units to be discriminated by the PLSDA (n° Y-Block); the number of latent vectors for each model (n° Latent Vectors-LV); and finally, the probability of random assignment of an individual into a category. RMSEC is the Root Mean Square Error of Calibration.

**PLSDA parameters**	**SIFT**	**RGBv+FD**
**n° classified elements**	3,099	4,800
**n° Y-block**	2	2
**n° LV**	18	10
**Cumulated Variance X-block (%)**	97.85	100
**Cumulated Variance Y-block (%)**	31.39	9.97
**Mean Specificity (%)**	100	100
**Mean Sensitivity (%)**	100	100
**Mean Class. Error (%)**	0	0
**Mean RMSEC**	0.50	0.51
**Random probability (%)**	50	50
**Mean Correct Classification Model (%)**	99.95	99.43
**Mean Correct Classification Independent Test (%)**	100	100

**Table 2.** Percentages of correct classification and number of different Type 1- and Type 2-Errors (see Section 2.3. for acronyms explications) obtained with the three different processing methods for the automated counting of squat lobsters (*Munida quadrispina*) at VENUS cabled observatory (see Figure 1). RGB, Red-Green-Blue colour channels; RGBv, Mean RGB; FD, Fourier Descriptors, PLSDA, Partial Least Square Discriminant Analysis; ED, Euclidean Distance; SIFT, Scale-Invariant Feature Transform.

	
	**% Correct Classification**	**Type-1**	**Type-2**
**RGBv+FD and PLSDA**	17	66	139
**SIFT and PLSDA**	51	51	11
**RGB+FD and ED**	40	91	0
